# Carbon Assimilation, Isotope Discrimination, Proline and Lipid Peroxidation Contribution to Barley (*Hordeum vulgare*) Salinity Tolerance

**DOI:** 10.3390/plants10020299

**Published:** 2021-02-04

**Authors:** Ioannis Vasilakoglou, Kico Dhima, Anastasia Giannakoula, Christos Dordas, Vasiliki Skiada, Kalliope Papadopoulou

**Affiliations:** 1 Department of Agriculture–Agrotechnology, University of Thessaly, 415 00 Larissa, Greece; vasilakoglou@uth.gr; 2 Department of Agriculture, International Hellenic University, 574 00 Echedoros, Greece; dimas@cp.teithe.gr; 3 Faculty of Agriculture, Forestry and Natural Environment, Aristotle University of Thessaloniki, 541 24 Thessaloniki, Greece; chdordas@agro.auth.gr; 4 Department of Biochemistry and Biotechnology, University of Thessaly, 415 00 Larissa, Greece; vask@bio.uth.gr (V.S.); kalpapad@bio.uth.gr (K.P.)

**Keywords:** carbon isotope discrimination, gene expression, hydrogen peroxide, lipid peroxidation

## Abstract

Barley (*Hordeum vulgare* L.) exhibits great adaptability to salt tolerance in marginal environments because of its great genetic diversity. Differences in main biochemical, physiological, and molecular processes, which could explain the different tolerance to soil salinity of 16 barley varieties, were examined during a two-year field experiment. The study was conducted in a saline soil with an electrical conductivity ranging from 7.3 to 11.5 dS/m. During the experiment, a number of different physiological and biochemical characteristics were evaluated when barley was at the two- to three-nodes growing stage (BBCH code 32–33). The results indicated that there were significant (*p* < 0.001) effects due to varieties for tolerance to salinity. Carbon isotopes discrimination was higher by 11.8% to 16.0% in salt tolerant varieties than that in the sensitive ones. Additionally, in the tolerant varieties, assimilation rates of CO_2_ and proline concentration were 200% and up to 67% higher than the sensitive varieties, respectively. However, in sensitive varieties, hydrogen peroxide and lipid peroxidation were enhanced, indicating an increased lipid peroxidation. The expression of the genes *Hsdr4*, *HvA1*, and *HvTX1* did not differ among barley varieties tested. This study suggests that the increased carbon isotopes discrimination, increased proline concentration (play an osmolyte source role), and decreased lipid peroxidation are traits that are associated with barley tolerance to soil salinity. Moreover, our findings that proline improves salt tolerance by up-regulating stress-protective enzymes and reducing oxidation of lipid membranes will encourage our hypothesis that there are specific mechanisms that can be co-related with the salt sensitivity or the tolerance of barley. Therefore, further research is needed to ensure the tolerance mechanisms that exclude NaCl in salt tolerant barley varieties and diminish accumulation of lipid peroxides through adaptive plant responses.

## 1. Introduction

Soil salinity is one of the prevailing edaphic factors in the environment that limit distribution and productivity of barley (*Hordeum vulgare*; 2n = 14; HH) [1,2]. Elevation of salinity levels in the soil causes many adverse effects on barley growth because of the low osmotic potential of soil solution [3]. Salt stress induces various biochemical and physiological responses in plants and affects most of plant metabolic processes [1,4]. Alterations in the integrity of cell membranes [5], as well as inhibition of different enzymatic activities and photosynthesis [6,7,8], are among the main processes affected. In addition, plants exposed to saline soils trigger generation of reactive oxygen species (ROS), which in turn have a negative oxidative stress effect on cellular structures and metabolism [9,10].

Generally, plants respond in various ways to stress. Osmotic stress caused by salinity is one of the major abiotic factors in Greece, limiting crop productivity, because it affects almost all plant functions. Proline is a well-known compatible solute that plays a central role in the process of osmotic adjustment in many plants. Most plant species can accumulate proline. According to [11], the amino acid proline (Pro), which widely occurs in higher plants, accumulates in large quantities in response to environmental stress.

Moreover, proline is considered to be involved in scavenging free radicals and in protecting enzymes in addition to their well-established roles as osmolytes. High concentrations of osmolytes, such as proline, can enhance the scavenging of reactive oxygen species (ROS) and response as antioxindants to oxidative stress [12].

Barley is an agronomically important crop, an important component of animal feed, which also provides health benefits in human diet. It is an important model organism for classical genetics; it has a diploid genome of 5.1 Gbp, consisting of seven chromosomes [13]. Barley exhibits small stomatal conductance, high maximum osmotic potential, vigorous growth, and high tillering ability, which contribute to salt tolerance in barley [14].

The use of stable isotope ratios of carbon for determination of the ratio of CO_2_ concentration in the leaf intercellular spaces to that in the atmosphere has emerged as an important tool that integrates physiological processes over time and provides useful information about the tolerance of plants to abiotic stress such as water stress and salinity [15]. The identification of responsive genes for biotic and abiotic stresses is a fundamental step in developing new tolerant varieties through conventional breeding [16]. Consequently, the physiological and biochemical processes, which are related to salinity tolerance, must be understood in order that better breeding tools for the evaluation of germplasm under salinity conditions (which are combined with drought) be found. This tolerant germplasm could significantly contribute to the increase of crop yields in soil with high saline concentration.

Barley is one of the most important cereal crops worldwide used for feed, malt, and human food. It has great adaptability to marginal environments experiencing drought, low temperatures, and salinity because of its great genetic diversity [14]. The barley genetic diversification and its adaptability to a broad range of ecological conditions have possibly highly strengthened its salinity tolerance, creating a rich gene pool with a large variation in adaptation to salinity [7]. The increase of barley tolerance to saline stress conditions would permit the use of problematic (saline) soils and regions and thus the increase of produced food and animal product. For this target, finding barley genetic material with a large capacity for tolerance to stress conditions of salinity, as well as the specification of biochemical and physiological processes that contribute to this tolerance, constitute a cutting edge point of agriculture [1,17]. However, most of the designed experiments and the analyzed data related to barley salinity tolerance have been conducted under controlled conditions (laboratory or greenhouse), failing to give extensive details for the salt tolerance under field conditions, while the related experiments conducted under saline field conditions are limited. The objective of this research was to determine differences in main biochemical (proline (Pro), hydrogen peroxide (H_2_O_2_), and lipid peroxidation), physiological (carbon assimilation rate, carbon composition, and isotope ratios) and molecular (gene expression) processes among 16 barley varieties, which have expressed different tolerance (sensitive to tolerant) to soil salinity under field conditions. This study compared the mechanisms that confer salinity tolerance among 16 barley varieties and explored the similarities or differences in their physiological mechanisms upon exposure to salt stress. It was hypothesized that there are specific mechanisms that can be co-related with the salt sensitivity or the tolerance of barley. Thus, this research can enhance our understanding of salinity tolerance mechanisms and will aid in the breeding of salt-tolerant barley plants.

## 2. Results

### 2.1. Barley Growth and Yield

The results indicated that effects due to salinity and variety were significant (*p* < 0.001) for barley total dry weight (TDW) and grain yield (GY) (Table 1).

**Figure 1:**
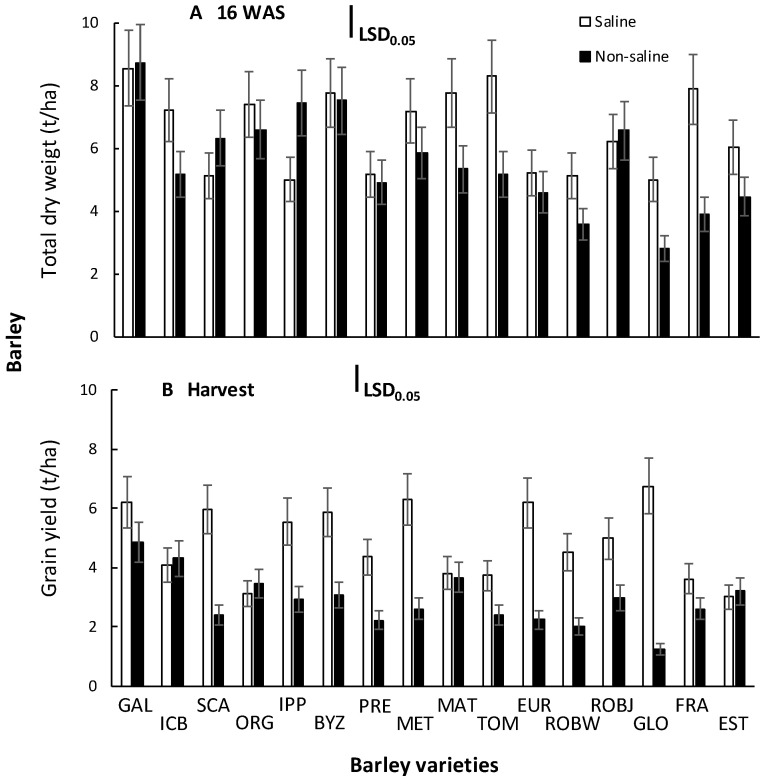
Total dry weight at 16 weeks after seeding (**A**), as well as grain yield at harvest (**B**) of 16 barley varieties grown in non-saline or in saline soil. Means are averaged across two years, and error bars denote the standard error of four biological replicates. GAL, Galt Brea ‘S’; ICB, ICB 100,126; SCA, Scarlet; ORG, Orgei/EH 165/Cross 270.2.3; IPP, Ippolytos; BYZ, Byzantio; PRE, Prestige; MET, Meteor; MAT, Matico ‘S’/LB Iran A-164; TOM, Tomillo ‘S’/DS4931 A-172; EUR, Europa; ROBW, Robur/WA 2196–68; ROBJ, Robur/J-126/OWB; GLO, 80.5060/Gloria ‘S’ A-196; FRA, Franka/6/Mona/Nopal ‘S’ A-242; EST, 4259/CI5831/Estate A-69.

No significant (*p* > 0.05) growing season *x* salinity *x* barley variety interaction were observed for barley TDW and GY. Thus, the means presented are averaged between the growing seasons (Figure 1).

**Figure 2:**
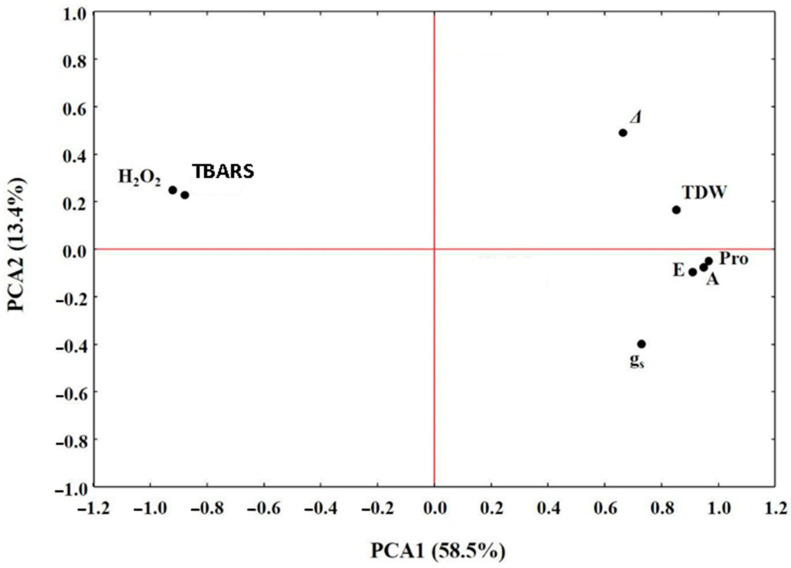
Principal components analysis (PCA) to assess the importance of barley traits contributing to soil salinity tolerance. TDW: total dry weight; Pro: proline concentration; A: carbon assimilation rate; E: transpiration rate; g_S_: stomatal conductance; H_2_O_2_: H_2_O_2_ content; TBARS: content; *Δ*: carbon isotope discrimination.

This study revealed that varieties Galt Brea ‘S’, Scarlet, Byzantio, Orgei/EH 165/Cross 270.2.3, and Robur/J-126/OWB provided the highest TDW of 6.20 to 9.04 t/ha at 16 WAS (Figure 1A). The lowest TDW was observed in the vars. 80.5060/Gloria ‘S’ A-196 and Robur/WA 2196–68 (2.82 and 3.60 t/ha, respectively). The principal components analysis (PCA) performed (Table 2) indicated that the first principal component (PCA1) accounted for 58.5%, while the second principal component (PCA2) accounted for 13.4% of the total variation (Figure 2).

The total dry weight was among the four most important traits contributing to barley salinity tolerance. At harvest, the highest grain yield was observed on vars. Galt Brea ‘S’ and ICB 100,126 (4.87 and 4.31 t/ha, respectively) (Figure 1B). These yields are about 19% and 5% lower, respectively, than those can achieved by these varieties under non-saline conditions. The barley var. Matico ‘S’/LB Iran A-164 provided sufficient yield under saline conditions, while the var. 80.5060/Gloria ‘S’ A-196 provided the lowest grain yield (1.25 t/ha), indicating the greatest sensitivity to saline. In fact, this variety was the most productive (6.74 t/ha) under non-saline conditions (Figure 1B).

### 2.2. Gas Exchange Measurements

**Figure 3:**
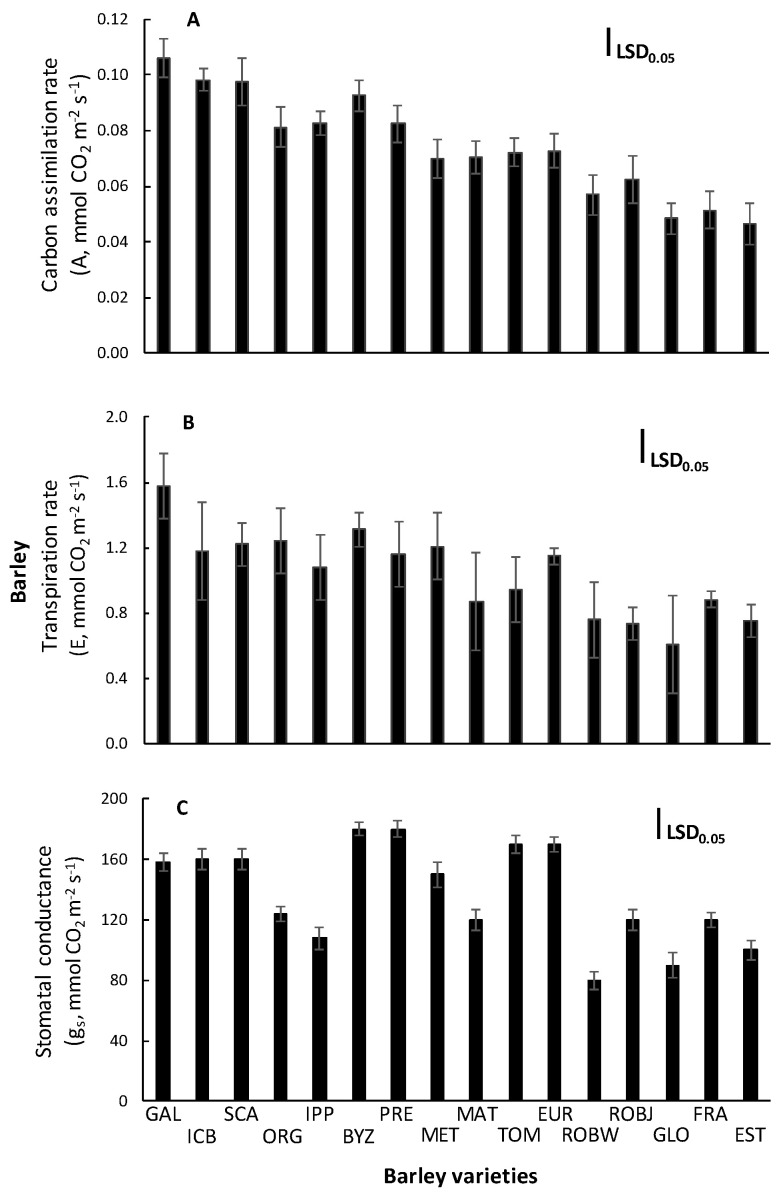
Carbon assimilation rate (A, μmol CO_2_ m^−^^2^ s^−1^) (**A**), transpiration rate (E, mmol H_2_O m^−2^ s^−1^) (**B**), and stomatal conductance (*g_s_*, mmol H_2_O m^−2^ s^−1^) (**C**) of the youngest fully expanded leaf of sixteen barley varieties grown in saline soil. Means are averaged across two years, and error bars denote the standard error of three biological replicates. GAL, Galt Brea ‘S’; ICB, ICB 100126; SCA, Scarlet; ORG, Orgei/EH 165/Cross 270.2.3; IPP, Ippolytos; BYZ, Byzantio; PRE, Prestige; MET, Meteor; MAT, Matico ‘S’/LB Iran A-164; TOM, Tomillo ‘S’/DS4931 A-172; EUR, Europa; ROBW, Robur/WA 2196–68; ROBJ, Robur/J-126/OWB; GLO, 80.5060/Gloria ‘S’ A-196; FRA, Franka/6/Mona/Nopal ‘S’ A-242; EST, 4259/CI5831/Estate A-69.

In order to estimate the effect of salinity on Calvin cycle, plant photosynthesis was measured. When tolerant varieties were compared to the sensitive ones, assimilation rate was markedly reduced by 50% to 55% under salinity stress in sensitive varieties (Figure 3A). A similar pattern was displayed by the transpiration rate (Figure 3B) and the stomatal conductance (Figure 3C), which were suppressed under salt. The PCA (Table 2) indicated that the A and E were also among the four most important traits contributing positively to barley salinity tolerance (Figure 2); however, this was not the case for the *g_s_*.

### 2.3. Carbon Isotope Discrimination (Δ)

**Figure 4:**
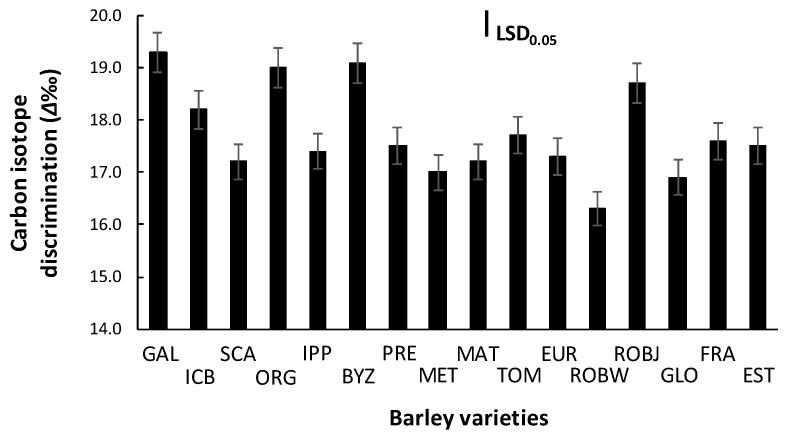
Carbon isotope discrimination (*Δ*) of sixteen barley varieties grown in saline soil. Means are averaged across two years, and error bars denote the standard error of three biological replicates. GAL, Galt Brea ‘S’; ICB, ICB 100,126; SCA, Scarlet; ORG, Orgei/EH 165/Cross 270.2.3; IPP, Ippolytos; BYZ, Byzantio; PRE, Prestige; MET, Meteor; MAT, Matico ‘S’/LB Iran A-164; TOM, Tomillo ‘S’/DS4931 A-172; EUR, Europa; ROBW, Robur/WA 2196–68; ROBJ, Robur/J-126/OWB; GLO, 80.5060/Gloria ‘S’ A-196; FRA, Franka/6/Mona/Nopal ‘S’ A-242; EST, 4259/CI5831/Estate A-69.

The ANOVA conducted for the carbon isotope data indicated that the *Δ* values differed among varieties (*p* < 0.001), most of the varieties provided higher dry matter, and grain yield showed, in most cases, higher *Δ* values (Figure 4).

The highest *Δ* values were detected in vars. Galt Brea ‘S’, Orgei/EH 165/Cross 270.2.3, Byzantio, and Robur/J-126/OWB, four of the eight varieties provided the greater yield in saline conditions. In contrast, the lowest values were observed on vars. Robur/WA 2196–68 and 80.5060/Gloria ‘S’ A-196, which also had the lowest total dry weight and grain yield. The PCA performed indicated that the *Δ* values contributed to barley salt tolerance, but not as much as the E and A traits (Figure 2).

### 2.4. Proline Determination

**Figure 5:**
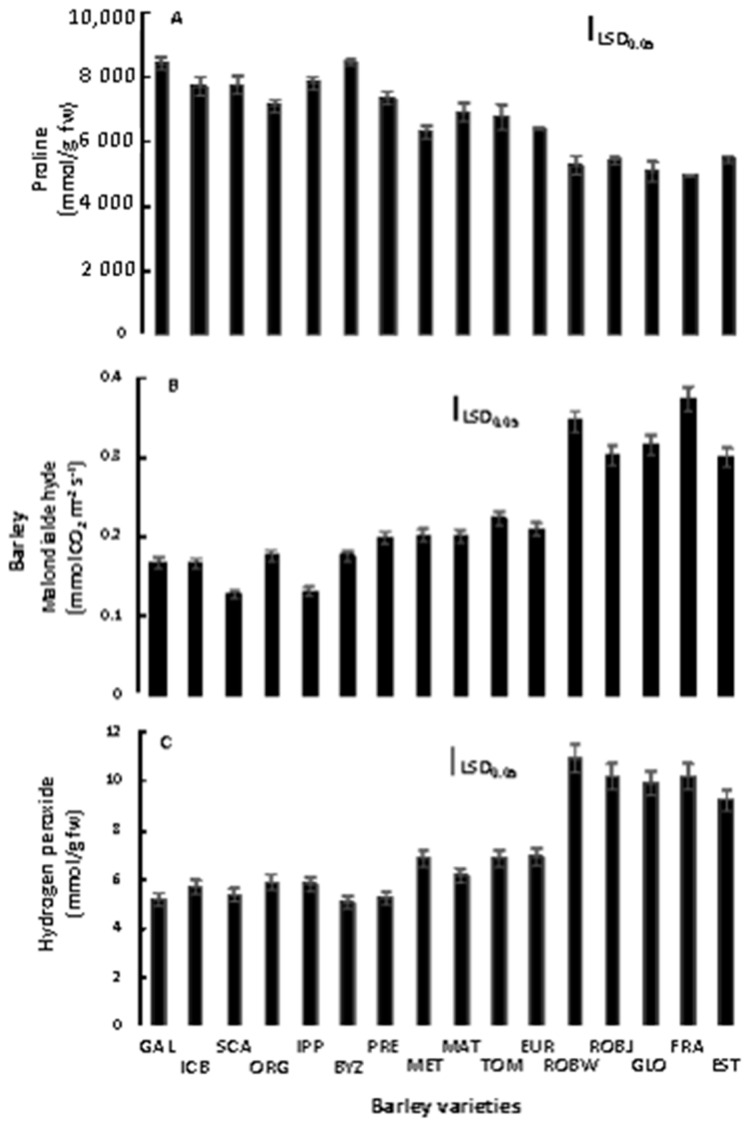
Proline (μmol g^−1^ fw) (**A**), malondialdehyde (nmol g^−1^ fw) (**B**), and hydrogen peroxide (mmol g^−1^ fw) (**C**) concentrations of sixteen barley varieties grown in saline soil. Means are averaged across two years, and error bars denote the standard error of three biological replicates. GAL, Galt Brea ‘S’; ICB, ICB 100126; SCA, Scarlet; ORG, Orgei/EH 165/Cross 270.2.3; IPP, Ippolytos; BYZ, Byzantio; PRE, Prestige; MET, Meteor; MAT, Matico ‘S’/LB Iran A-164; TOM, Tomillo ‘S’/DS4931 A-172; EUR, Europa; ROBW, Robur/WA 2196–68; ROBJ, Robur/J-126/OWB; GLO, 80.5060/Gloria ‘S’ A-196; FRA, Franka/6/Mona/Nopal ‘S’ A-242; EST, 4259/CI5831/Estate A-69.

Concentrations of proline in barley varieties grown under salinity are presented in Figure 5A. In particular, Pro accumulation in the tolerant to salinity variety Galt Brea ‘S’ was about 67% greater than that in the sensitive variety 80.5060/Gloria ‘S’ A-196. The PCA indicated that the Pro accumulation was the most important trait positively contributing to barley salinity tolerance (Figure 2). Additionally, the PCA indicated that the E, A, and Pro concentration traits are strongly correlated with each other.

### 2.5. Lipid Peroxidation and Hydrogen Peroxide Assays

Under salinity conditions, significant increases in concentration of MDA in barley sensitive vars. were observed (Figure 5B). TBARs content in the sensitive varieties were about 2.5-times higher than those in the tolerant ones, and from the PCA analysis (Table 2), it was noted that TBARs content negatively contributed to barley salinity tolerance (Figure 2).

A similar trend for H_2_O_2_ was observed in the barley varieties evaluated (Figure 5C). The H_2_O_2_ values in the sensitive varieties were up to two times higher than those in the tolerant ones. In this study, it was clear from PCA analysis that there was significant negative correlation between barley salinity tolerance and contents of H_2_O_2_ in the barley plants (Figure 2). This study demonstrated that the magnitude of both TBARs and H_2_O_2_ content increase in barley plants that are sensitive to high concentration of salt in soil, but influence E, A, and Pro traits negatively.

### 2.6. Gene Expression

**Figure 6:**
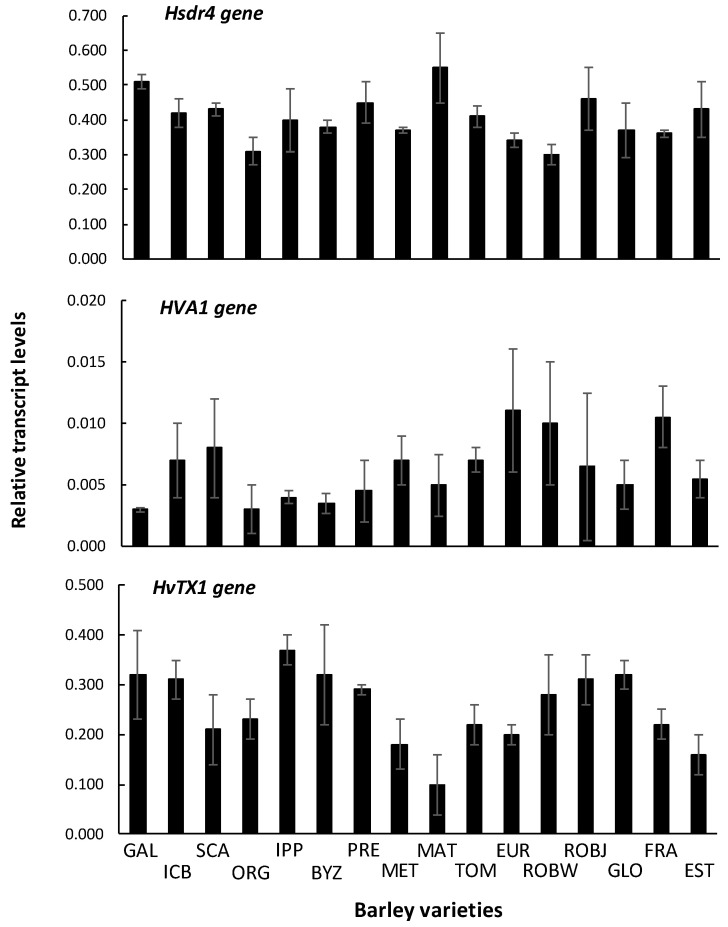
Gene transcript levels of barley drought inducible genes *Hsdr4*, *HvA1*, and *HvTX1* in leaf tissues. Error bars denote the standard error of three biological replicates. GAL: Galt Brea ‘S’; ICB: ICB 100,126; SCA: Scarlet; ORG: Orgei/EH 165/Cross 270.2.3; IPP: Ippolytos; BYZ: Byzantio; PRE: Prestige; MET: Meteor; MAT: Matico ‘S’/LB Iran A-164; TOM: Tomillo ‘S’/DS4931 A-172; EUR: Europa; ROBW: Robur/WA 2196–68; ROBJ: Robur/J-126/OWB; GLO: 80.5060/Gloria ‘S’ A-196; FRA: Franka/6/Mona/Nopal ‘S’ A-242; EST: 4259/CI5831/Estate A-69.

Expression levels of Hsdr4 gene showed no differences among the genotypes examined. Expression levels of HvA1 gene were lower in some tolerant and intermediate tolerant varieties (GAL, ORG, IPP, BYZ, and PRE). All other varieties demonstrated higher levels of HvA1 expression, though differences among cultivars were not statistically significant (Figure 6). Expression levels of HvTX1 gene showed similar expression levels among all varieties examined; only MAT variety demonstrated lower transcript levels in comparison to the other genotypes, though differences were not statistically significant. Statistical analyses comparing each gene expression values among genotypes showed no significant differences (one way ANOVA, 0.05 level). When values of biological replicates of the four broader categories of cultivars (tolerant, intermediate tolerant, intermediate sensitive, sensitive) were averaged and compared, statistical analyses showed no significant differences (one-way ANOVA, 0.05 level). When values among tolerant and sensitive varieties were averaged and compared, statistical analyses showed no significant differences (Student’s *t*-test, 0.05 level).

## 3. Discussion

### 3.1. Barley Growth and Yield

Despite the fact that barley can tolerate medium levels of NaCl, its growth is adversely affected to a certain extent by salinity, owing to Na+ interference in K^+^ and Ca^2+^ assimilation [18]. Two of the barley varieties studied had the ability to restrict the negative effect of salinity. The grain yield reduction in the other varieties was up to 81.5% (based on grain yield which can be achieved under non-saline conditions). Barley that is grown in saline soil experiences yield reduction of 28% to 45% [19]. It has also been found [20] that salt susceptible varieties grown under salt stress conditions of about 200 mM NaCl reduce barley grain yield from 47.5% to 79.6%.

### 3.2. Gas Exchange Measurements

Assimilation rate, transpiration rate, and stomatal conductance were reduced under salinity stress conditions, mainly in the sensitive varieties, due to their effort to close the stomata and to inhibit the water losses. Additionally, the PCA indicated that the A and E were also among the four most important traits contributing positively to barley salinity tolerance, while this was not the case for the *g_s_*. The tolerant barley varieties reduced water losses, adjusting their osmotic potential, and achieved satisfactory photosynthesis rate by maintaining the stomata partly open. According to [21], no significant correlation between salinity tolerance and stomatal conductance was found, but the increase of stomatal density in barley had as result the minimization of the non-stomatal transpiration. These characteristics could lead to increased water use efficiency and increased salinity tolerance. Additionally, [22] found no association between stomatal conductance and barley salinity tolerance; however, [23] found positive correlation between salinity stress tolerances and leaf stomatal conductance in wheat (*Triticum aestivum* L.). In our study, the low CO_2_ availability measured by stomatal conductance in the two most sensitive varieties (vars. 80.5060/Gloria ‘S’ A-196 and Robur/WA 2196–68) negatively affects net photosynthesis under salinity.

### 3.3. Carbon Isotope Discrimination (Δ)

Soil salinity caused a significant variability in *Δ* in barley varieties, indicating that salt stress can lead to less discrimination against the heavier carbon isotope. Most of the varieties provided high dry matter and grain yield showed high *Δ* values. Generally, a higher ratio (Ci/Ca) of intercellular (Ci) to atmospheric (Ca) of CO_2_ leads to higher *Δ*, mainly because of smaller stomatal conductance [24]. This fact has as a result higher assimilation rate and therefore higher yield. In C_3_ plant leaves, like barley, photosynthetic gas exchange is related to *Δ*, which is in part determined by the ratio of CO_2_ concentration in the leaf intercellular spaces (C_i_) to that in the atmosphere (C_a_) (C_i_/C_a_) [15]. Variation in stomatal opening affects the supply rate of CO_2_, and in chloroplast, demand for CO_2_ differentiates the *Δ* values among plants.

Wheat and barley grown in soil with high concentration of salt have shown a decrease in *Δ* [25,26]. In this study, PCA indicated that the *Δ* values contributed to the expression of genes for salt tolerance in barley; however, alteration of the *Δ* in the leaf tissues due to stress gives information about the way the plant succeeds in maintaining water use efficacy [27]. In this study, the genetic variation found in barley for *Δ* could be due to the differences in photosynthetic capacity of the different varieties rather than in stomatal conductance alone under the influence of salinity. Under Mediterranean conditions, significant genotypic variation for *Δ* of wheat and barley flag leaves has been detected [28].

### 3.4. Proline Determination

The PCA indicated that the Pro accumulation was the most important trait positively contributing to barley salinity tolerance. Generally, Pro is a very important plant osmolyte, but the mechanisms of Pro action are not fully understood. Previous reasearchers [29] found that the salt-tolerant barley accumulated more compatible solutes such as Pro and inositol, acquiring high antioxidant ability to cope with ROS, and consuming less energy under salt stress for producing biomass. In addition, Ref. [30] found that increased Pro was associated with stress tolerance, while [31] have reported that, under stress conditions, Pro contributes to stabilizing sub-cellular structures, such as membranes and proteins, scavenging free radicals and buffering cellular redox potential.

### 3.5. Lipid Peroxidation and Hydrogen Peroxide Assays

Under salinity conditions, significant increases in TBARs concentration in barley sensitive varieties were observed. Similar results have been reported by [32], who found that salt treatment caused an increase of lipid peroxidation revealed by high TBARs content in sensitive barley genotypes, but no significant effect was shown in the most salt-tolerant genotypes. Additionally, the increase of TBARs content has been reported in barley plants grown under high-salt stress [30]. Regarding the biochemical role of MDA, it is one of the decomposition products of polyunsaturated fatty acids of biomembranes. Additionally, it has frequently been used as a biomarker for lipid peroxidation and cell membrane and ultrastructure damages.

In the current experiment, salinity induced the enhancement of lipid peroxidation in the sensitive barley varieties. On the contrary, the tolerant varieties limited lipid peroxidation during salinity stress. This enhancement of H_2_O_2_ and TBARs content indicates the prevalence of oxidative stress, and this may be one of the possible mechanisms by which toxicity of salinity could be manifested in the plant tissues [33,34]. As a result, low H_2_O_2_ content is strongly correlated with tolerance to salt-induced oxidative stress in wheat and barley [34]. Based on these observations, salinity tolerance of barley varieties could be attributed to a mechanism that minimizes the accumulation of lipid peroxides. Thus, barley salinity tolerance could be increased by an increased antioxidant system activity in order to limit cellular damage.

### 3.6. Gene Expression

So far, not many studies have been applied to target the responses of genes to a salinity stress, or to detect the responsible genes for salinity tolerance in barley. Under a high number of abiotic stresses, for instance heat, drought, and low temperature, genes were previously found to be responsive to salinity stress [35,36]. The *Hsdr4* gene (*Hordeum spontaneum* dehydration-responsive 4) has a role in plant tolerance to dehydration stress, since it has been previously observed to be differentially expressed in sensitive versus tolerant wild barley (*H. spontaneum*) genotypes when these are exposed to dehydration stress [37]. Notably, *Hsdr4* gene is considered as water stress inducible, and can thus be used when assessing barley varieties based on their tolerance to water deficiency abiotic stress. Results of this study demonstrate that *Hsdr4* gene shows no differences among genotypes of barley examined. This may be attributed to the relatively late stage of sampling.

Additionally, Ref. [16] characterized the barley *HvTX1* gene (TRX-like H3K4 methyltransferase) as the homologue to *Arabidopsis thaliana ATX1*/*ATX2* genes and studied its involvement in drought stress. Furthermore, the *HvA1* is a barley group III Late Embryogenesis Abundant (LEA) protein. The LEA proteins are ubiquitous in plants and are produced in response to dehydration stress. They contribute in water status stabilization, protection of cytosolic structures, ion sequestration, protein renaturation, transport of nuclear targeted proteins, prevention of membrane leakage, and membrane and protein stabilization. They have been considered to play a key role in water-deficit tolerance [38].

Based on the above mentioned, the expression levels of the under-study genes in saline tolerant varieties (provided the greatest yield in saline conditions) were expected to be higher in comparison to intermediate tolerant or sensitive ones. According to [16], expression levels may be dependent on drought exposure time and/or the developmental stage of the plant. However, Ref. [39] employed the constitutive and high-level expression of *HvA1* gene in transgenic rice plants, in order to assess the role of the *HvA1* protein under water and salt-stress conditions, and found that transgenic plants exhibited significantly increased tolerance when exposed to the above mentioned abiotic stresses. Additionally, transgenic wheat plant lines constitutively expressing the *HvA1* gene exhibited an improvement in important agronomic traits such as total dry mass and water use efficiency, in comparison to controls, when these plants were grown under soil water deficit conditions [40]. Ref. [41] recorded an induction in *HvA1* gene expression levels in barley seedlings when these were exposed to a variety of treatments, with drought and salt stress being among them. Ref. [42] produced *HvA1* transgenic rice (*Oryza sativa* L.) plants by using a promoter that keeps low basal level of *HvA1* under normal growth conditions, but confers a high level of expression upon induction by drought, salt, and cold stresses. Additionally, Ref. [43] found that the expression of abiotic stress-related genes *HvPIP1.3*, *DREB2*, and *DWARF4* in barley increased after salt treatment, while the expression of *HvPIP1.2* and *WAK* reduced.

Recently, an investigation related to the effect of 5-aminolevulinic acid (ALA) on salinity tolerance of six barley genotypes showed that ALA induced pigment contents, antioxidants enzymes activity, and stress-responsive genes expression, relative to NaCl-stressed plants [44].

Absence of differences in expression of drought/salt stress associated genes among varieties can be attributed to the progressed time of tissue sampling, where tolerance to salt-stress factor might have already been conferred. Perhaps sampling at earlier stages of plant development would show differences at transcript levels of these genes among the barley varieties examined.

Besides the processes mentioned above, there are other possible mechanisms that are involved in salinity tolerance. Ref. [45] found that, in the sensitive barley variety *Triumph* grown in NaCl, the mean cytoplasmic Na concentration was about 1.4 times greater than that in the tolerant barley variety, while the cytoplasmic K concentration in tolerant variety was reduced less than in the sensitive one.

## 4. Materials and Methods

### 4.1. Experimental Site

**Figure 7:**
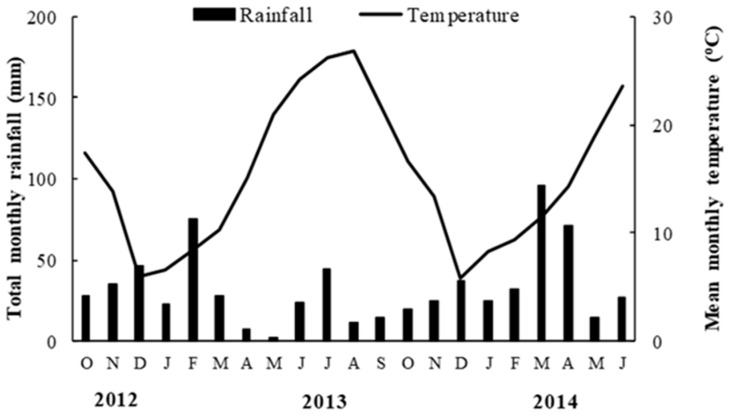
Weather data (mean monthly temperature and monthly rainfall) during the field experiment.

A two-year field experiment was conducted during the 2012/13 and 2013/14 growing season to investigate barley tolerance to soil salinity. The experiment was located at the Technological Educational Institute Farm of Thessaloniki in Northern Greece (22°48′33″ E and 40°39′09″ N; elevation 0 m), an area that has predominantly saline, sandy loam (Typic Xeropsamment) soil. The characteristics of the saline soil were clay at 56 g/kg, silt at 180 g/kg, sand at 764 g/kg, organic matter at 9 g/kg, potassium at 57.2 mg/kg, sodium at 3130.6 mg/kg, and soil pH at 8.1(1:2 H_2_O). In both years, the soil analysis conducted in early October showed that the initial soil electrical conductivity in the field ranged from 7.3 to 11.5 dS/m (averaged 9.4 dS/m across samples). Simultaneously, in order to be assessed the barley growth and yield under non-saline conditions, barley varieties had been cultivated in a non-saline soil (sodium at 394 mg/kg and electrical conductivity at 0.4 dS/m) inside the same farm. The weather data (mean monthly temperature and total monthly rainfall) are presented in Figure 7.

#### 4.1.1. Genotypes

Sixteen barley varieties, selected in a greenhouse experiment evaluating barley tolerance to salinity, were used in the experiment. In particular, three varieties (Galt Brea ‘S’ (GAL), ICB 100,126 (ICB), and Scarlet (SCA)) were rated as tolerant to soil salinity, according to their tillering ability, biomass, and seed yield achieved in saline conditions. Additionally, five varieties (Orgei/EH 165/Cross 270.2.3 (ORG), Ippolytos (IPP), Byzantio (BYZ), Prestige (PRE), and Meteor (MET)) were rated as intermediate tolerant to soil salinity, two varieties (Matico ‘S’/LB Iran A-164 (MAT) and Tomillo ‘S’/DS4931 A-172 (TOM)) were rated as intermediate sensitive, and six varieties (Europa (EUR), Robur/WA 2196–68 (ROBW), Robur/J-126/OWB (ROBJ), 80.5060/Gloria ‘S’ A-196 (GLO), Franka/6/Mona/Nopal ‘S’ A-242 (FRA), and 4259/CI5831/Estate A-69 (EST)) were rated as sensitive.

#### 4.1.2. Experimental Procedure

The experimental area was cultivated in early November with a mouldboard plough, while a harrow disk was used for fertilizer incorporation. Barley varieties were hand-seeded in a plot size measuring 3 × 2 m with inter-row spacing of 16 cm at a seeding rate of 200 kg/ha (approximately 250 plants/m^2^). All plots were separated by 1-m wide alleys. Additionally, blocks were separated by 2-m wide alleyways. The seeding dates were 12 November 2012, and 20 November 2013. At seeding time, nitrogen and phosphorus, applied as ammonium sulpho-phosphate (20-10-0) at 100 and 50 kg/ha, respectively, were incorporated into the soil. An additional 50 kg N/ha as ammonium nitrate (33.5-0-0) was applied when barley plants attained one- to two-node growth stage (BBCH code 31–32) [46].

A randomized complete block design with four replicates was used. In each plot, 12 rows of one of the 16 barley varieties were included. No irrigation was applied during the experiment.

#### 4.1.3. Data Collection

Each plot was harvested 16 weeks after seeding (WAS), when barley plants attained two- to three-node growth stage (BBCH code 32–33). Samples from each plot were hand-harvested from two 2-m long rows, which constituted an area of 0.64 m^2^. Harvesting was done by cutting the plants at the base and drying in an oven maintained at 70 °C for three days before determining the total dry weight. Barley harvest was conducted on 20 June 2013, and on 25 June 2014. In all plots, barley plants were hand-harvested from seven 1-m long rows, which constituted an area of 1 m^2^. Grain weight of these plants was used for the determination of barley grain yield.

In both years, different physiological and biochemical measurements were used to evaluate the reaction of barley varieties on saline stress. In particular, gas exchange measurements (carbon assimilation rate (A), transpiration rate (E), intercellular concentration of CO_2_, and stomatal conductance (g_S_)) and carbon composition and isotope ratios, as well as proline (Pro) concentration, hydrogen peroxide (H_2_O_2_), and lipid peroxidation levels were determined. Furthermore, RNA isolation, cDNA synthesis, and gene expression analysis were used to explain barley germplasm variability in soil salinity tolerance.

### 4.2. Gas Exchange Measurements

Five measurements of gas exchange were conducted in each plot at 16 WAS on the youngest fully expanded barley leaf at growth stage of middle of heading (BBCH code 55) with an IRGA Li-6400 portable photosynthesis meter (LiCor, Inc. Lincoln, NE, USA). Calculations of carbon assimilation rate (A), transpiration rate (E), intercellular concentration of CO_2_, and stomatal conductance (*g_S_*) from gas exchange measurements were according to [47].

### 4.3. Carbon Isotope Discrimination (Δ)

A sample of 20 flag leaves (known as the most essential organ for photosynthesis in C3 plants) per plot were collected during the grain filling stage (BBCH code 71–73) and placed in paper bags and dried in an oven maintained at 70 °C for three days. The dried samples were ground in a Wiley mill through a 1-mm screen, weighted, and placed directly into tin capsules for isotope analysis. An isotope mass spectrometer (CF-IRMS, PDZ-Europa, Crewe, UK) coupled with an elemental analyzer for an on-line sample preparation was used for the carbon (C) composition and isotope ratios in leaf samples measurement. Additionally, the following equation was used for natural abundance of carbon (δ^13C^) isotopes determination [15]:

δ^C13^ (‰) = (R_leaf_ − R_standard_)/R_standard_

where R_leaf_ was the ^13^C/^12^C ratio in plant tissue and R_standard_ was the respective ratio of the standard. For the *C* isotope, the Pee Dee Belemnite (PDB) limestone, which is the universally accepted standard, was used. Furthermore, the following equation was used for the carbon isotope discrimination (*Δ*, ‰) calculation:

*Δ* = δ_air_ − δ_leaf_/(1 + δ_leaf_)

where, δ_air_ and δ_leaf_ were δ^13C^ of the air and leaf sample, respectively (δ^air^ is ca −8‰).

### 4.4. Determination of Proline

Sampled barley fully developed young leaves at 16 WAS of 10 collected plants from each plot were cut into small pieces. From these samples, approximately 0.3 g were weighed and placed separately in glass vials containing 10 mL of 80% (*v*/*v*) ethanol. Then, they were heated at 60 °C for 30 min, and the extracts were filtered and diluted with 80% (*v*/*v*) ethanol up to 20 mL. In these barley extracts, the free Pro concentration was determined by the acid-ninhydrin reagent method [48]. Approximately 1 g ninhydrin was added to 500 mL of dense H_2_SO_4_. Then, 2 mL of the acid-ninhydrin and 2 mL of the aqueous alcohol extract were transferred into test tubes. The test tubes were covered by glass marbles to minimize evaporation and maintained at 95 °C for 60 min in a water bath. After this time, they were allowed to cool at room temperature. Finally, 4 mL of toluene was added to each replicate of the sample and mixed thoroughly. After separation of solution layers, the toluene layer was carefully decanted, placed in glass corvettes, and its absorption was determined at 518 nm.

### 4.5. Lipid Peroxidation and Hydrogen Peroxide Assays

The level of lipid peroxidation in barley fully developed young barley leaves was measured as TBARs content. The TBARs content was determined by reaction with 2-thiobarbituric acid (TBA)-reactive substances, as described by [49]. Samples of barley leaf tissues were homogenized at 4 °C, in a TBA solution comprised of 20.0% (*w*/*v*) trichloroacetic acid and 0.01% butylated hydroxytoluene. The solution absorbance was evaluated at 440, 532, and 600 nm wavelength. Then, the TBARs concentration was calculated as the difference of absorbance at 440, 532, and 600 nm wavelength, using the extinction coefficient 157 mmol^−1^ cm^−1^, and was expressed as nmol mda/g fresh weight.

Hydrogen peroxide (H_2_O_2_) content in the fully developed young leaves at 16 WAS was determined using the method described by [50]. Potassium iodide (KI) was used to determine the H_2_O_2_ in the leaf samples at 390 nm and for 1 h in darkness. Regarding the reaction mixture of barley leaf extract supernatant, it consisted of 2 mL of reagent (1 M KI *w/v* in double-distilled water), 0.5 mL of 100 mM K-phosphate buffer, and 0.5 mL of 0.1% trichloroacetic acid (TCA). Additionally, 0.1% TCA in the absence of barley leaf extract consisted the blank probe. A known H_2_O_2_ concentrations was used for the standardization of the H_2_O_2_ content.

### 4.6. RNA Isolation and cDNA Synthesis

Barley leaf tissues, taken at 16 WAS and stored in liquid nitrogen, were grounded with a pestle under the presence of liquid nitrogen and total DNA was isolated using the CTAB method [51]. Total RNA was extracted using a phenol:chloroform extraction and LiCl precipitation protocol. To eliminate genomic DNA carry over in subsequent reactions, samples were treated with DNase I (Invitrogen) at 37 °C for 1 h. Subsequently, primers specific for barley a-tubulin gene, a-tubulinF (5′- TCCATGATGGCCAAGTGTGA-3′), and a-tubulinR (5′-CTCATGTACCGTGGGGATGTC-3′) [48] were used in PCR in order to verify the complete DNA removal, using barley genomic DNA as a positive control. First strand cDNA synthesis was performed in circa 0.6 μg of DNase treated total RNA, using the SuperScript^®^ II Reverse Transcriptase (Invitrogen) according to the manufacturer’s instructions. The resulting cDNA was normalized for the expression of the housekeeping gene *ADP* (ADP-ribosylation factor 1) using the RT-PCR primers ADP-F (5′-CGTGACGCTGTGTTGCTTGT-3′) and ADP-R (5′-CCGCATTCATCGCATTAGG-3′) [49].

### 4.7. Analyses of Gene Expression

For gene expression data analysis, we used a variation of the equation [52] and the −*ΔΔ*Ct method described by [52]. More specifically we used the *Δ*Cq approach using a reference gene, taking into account the fact that the amplification efficiencies of the two amplicons (target and reference gene) are not the same. This method uses the difference between the reference and target Cq values for each sample. Normalization of the gene expression was performed by using a reference (housekeeping gene, ADP gene in our case). Τhe equation used for gene expression calculations becomes as follows:

$$ratio\left(reference/target\right)=\frac{E_{ref}{}^{Cq_{ref}}}{E_{target}{}^{Cq_{target}}}$$

where,

*E_ref_* is the real-time PCR efficiency of the reference gene transcript

*E_target_* is the real-time PCR efficiency of target gene transcript

*Cq_ref_* is the quantification cycle of the reference gene transcript

*Cq_target_* is the quantification cycle of the target gene transcript.

Gene expression analysis was performed in order to assess the under-study barley varieties for their potential tolerance to salinity. Quantitative real-time PCR (Q-PCR) analyses were performed on an MX 3005P system (Stratagene) using the SYBR FAST qPCR Kit (KAPABIOSYSTEMS) and gene-specific primers, following instructions of the manufacturer. Each genotype was assessed in three biological replicates. A 254 bp sequence fragment of barley *HvTX1* gene [16] was targeted with the primers F3 (5′-TGGATAAGCAGGGATACGATGAGC-3′) and R2 (5′-TCAGAAAGGGCACCGCCTGCTTC-3′). Furthermore, a circa 70 bp fragment of barley *Hsdr4* gene was targeted with the primers Hsdr4F (5′-CCGGGCTTTATTCCTGGCT-3′) and Hsdr4R (5′-TTTCCAGTACAACCCTCCGCT-3′) [50]. Additionally, a 197 bp sequence fragment of barley *HvA1* gene was targeted with the primers HVA1F (5′-GCGCAGTACACCAAGGAGTC-3′) and HVA1R (5′-GTGGTGGTGGTGTCCTTGAC-3′).

The Q-PCR conditions were set as follows: initial denaturation at 95 °C for 3 min, followed by 40 cycles of 95 °C for 15 s, 60 °C for 20 s, and 72 °C for 11 s. The primer specificity and the formation of primer dimers were monitored by dissociation curve analysis. In all samples, a single amplicon was detected, and relative abundance of all transcripts amplified was normalized to the expression level of barley *ADP* gene. The latter was selected as internal reference gene due to its stability not only under salinity stress (stable expression in control and salinity-treated barley plants), but also when the developmental stage of the plant was taken into consideration [53]. Data were analyzed according to [54], taking into account the fact that the amplification efficiencies of the two amplicons (target and reference gene) are not the same. The reaction efficiencies were estimated via the computer program LinRegPCR [55].

### 4.8. Statistical Analyses

An over year analysis of variance (ANOVA) was used for the data obtained from the field experiment (total dry weight and grain yield). The ANOVA and the homogeneity of variances was examined with the Bartlett’s test using the Statistical Package for the Social Sciences (SPSS, 2006) program. Additionally, the SPSS program was used for the conduction of the principal components analysis (PCA), including the barley total dry weight at 16 WAS, Pro concentration, carbon assimilation rate (A), transpiration rate (E), stomatal conductance (*g_S_*), H_2_O_2_ content, MDA content, and *Δ*. The PCA was performed to determine the most important traits contributing to barley salinity tolerance and if genes tested were correlated with barley tolerance to salinity [7]. The Fisher’s Protected Least Significant Difference test procedures were used to detect and separate mean treatment differences at *p* = 0.05.

For gene expression analysis, one way ANOVA was used in comparisons among individual genotypes (0.05 level), and among the four broader categories of barley varieties (tolerant, intermediate tolerant, intermediate sensitive, sensitive). Student’s *t*-test was used to compare relative expression values among average values of biological replicated of tolerant (tolerant and intermediate tolerant) versus sensitive (intermediate sensitive and sensitive) cultivars (0.05 level).

## 5. Conclusions

The results of this study indicated that there was a great variability among barley varieties regarding their tolerance to soil salinity. The barley varieties that achieved the greatest growth and yield in field saline conditions showed greater carbon assimilation rate compared with the sensitive ones. The proline concentration was positively correlated with the tolerance to soil salinity, while the TBARs and hydrogen peroxide were negatively correlated with the salinity tolerance. This fact indicates that the prevalence of oxidative stress could be one of the possible mechanisms by which salinity toxicity in the barley tissues is manifested. That is, enhancement of lipid peroxidation in the sensitive barley varieties was triggered by salinity, while lipid peroxidation during salinity stress in the tolerant barley varieties was lower. Based on these observations, salinity tolerance of the barley varieties could be associated with a mechanism that minimizes the accumulation of lipid peroxides. Thus, the ability to increase the antioxidant system activity in order to limit cellular damage might be an important attribute linked to salinity tolerance in barley. No differences in expression of drought/salt stress associated genes (*HvTX1*, *Hsdr4,* and *HvA1*) were observed among varieties tested. However, there is great genetic variation, both within and between species, in the resistance of plants to salinity, suggesting that salinity-tolerant species or varieties possess several mechanisms for detoxifying salinity. Therefore further investigating is required to certify genetic variability and adaptive mechanisms of barley for enhancing salt tolerance and crop productivity.

## Figures and Tables

**Table 1:** Analysis of variance for the total dry weight (TDW) and gain yield (GY) data of 16 barley varieties as affected by year and soil salinity.

		Significance of *F* Ratio ^a^	
Source	df	TDW	GY
Year (Y)	1	NS	NS
Salinity (S)	1	***	***
YS	1	NS	NS
R(YS)	12	***	**
Variety (V)	15	***	***
YV	15	NS	NS
SV	15	***	***
YSV	15	NS	NS
Error	180		
CV, %		14.6	16.2

^a^, ** or ***, significant at the 0.01 or 0.001 level, respectively; NS, not significant.

**Table 2:** Correlation matrix of the principal component analysis performed for the barley traits ^a^.

Correlation	TDW	GY	*Δ*	A	E	g
TDW	1.000	0.695	0.789	0.757	0.792	0.535
GY	0.695	1.000	0.679	0.575	0.554	0.218
*Δ*	0.789	0.679	1.000	0.534	0.548	0.402
A	0.757	0.575	0.534	1.000	0.883	0.689
E	0.792	0.554	0.548	0.883	1.000	0.714
g	0.535	0.218	0.402	0.689	0.714	1.000
TDW		0.001	0.000	0.000	0.000	0.016
GY	0.001		0.002	0.010	0.013	0.208
*Δ*	0.000	0.002		0.017	0.014	0.061
A	0.000	0.010	0.017		0.000	0.002
E	0.000	0.013	0.014	0.000		0.001
g	0.016	0.208	0.061	0.002	0.001	

^a^ TDW, total dry weight; GY, grain yield.

## Data Availability

Not applicable.

## Abbreviations

GY	grain yield
KI	potassium iodide
MDA	malondialdehyde
PCA	principal component analysis
Pro	Proline
TCA	trichloroacetic acid
TDW	total dry weight
WAS	weeks after seeding
